# Treatment of Fabry Disease: Outcome of a Comparative Trial with Agalsidase Alfa or Beta at a Dose of 0.2 mg/kg

**DOI:** 10.1371/journal.pone.0000598

**Published:** 2007-07-11

**Authors:** Anouk C. Vedder, Gabor E. Linthorst, Gunnar Houge, Johannna E.M. Groener, Els E. Ormel, Berto J. Bouma, Johannes M.F.G. Aerts, Asle Hirth, Carla E.M. Hollak

**Affiliations:** 1 Department of Internal Medicine/Endocrinology and Metabolism, Academic Medical Center, Amsterdam, The Netherlands; 2 Department of Medical Biochemistry, Academic Medical Center/University of Amsterdam, Amsterdam, The Netherlands; 3 Center for Medical Genetics and Molecular Medicine, Haukeland University Hospital, Bergen, Norway; 4 Department of Cardiology, Academic Medical Center, Amsterdam, The Netherlands; 5 Department of Heart Disease, Haukeland University Hospital, Bergen, Norway; National Institutes of Health, United States of America

## Abstract

**Background:**

Two different enzyme preparations, agalsidase alfa (Replagal^TM^, Shire) and beta (Fabrazyme^TM^, Genzyme), are registered for treatment of Fabry disease. We compared the efficacy of and tolerability towards the two agalsidase preparations administered at identical protein dose in a randomized controlled open label trial.

**Methodology/Principal Findings:**

Thirty-four Fabry disease patients were treated with either agalsidase alfa or agalsidase beta at equal dose of 0.2 mg/kg biweekly. Primary endpoint was reduction in left ventricular mass after 12 and 24 months of treatment. Other endpoints included occurrence of treatment failure (defined as progression of cardiac, renal or cerebral disease), glomerular filtration rate, pain, anti-agalsidase antibodies, and globotriaosylceramide levels in plasma and urine. After 12 and 24 months of treatment no reduction in left ventricular mass was seen, which was not different between the two treatment groups. Also, no differences in glomerular filtration rate, pain and decline in globotriaosylceramide levels were found. Antibodies developed only in males (4/8 in the agalsidase alfa group and 6/8 in the agalsidase beta group). Treatment failure within 24 months of therapy was seen in 8/34 patients: 6 male patients (3 in each treatment group) and 2 female patients (both agalsidase alfa). The occurrence of treatment failures did not differ between the two treatment groups; χ^2^ = 0.38 p = 0.54.

**Conclusion:**

Our study revealed no difference in reduction of left ventricular mass or other disease parameters after 12 and 24 months of treatment with either agalsidase alfa or beta at a dose of 0.2 mg/kg biweekly. Treatment failure occurred frequently in both groups and seems related to age and severe pre-treatment disease.

**Trial Registration:**

International Standard Randomized Clinical Trial ISRCTN45178534

## Introduction

Fabry disease is an X-linked lysosomal storage disorder caused by the deficiency of the lysosomal enzyme α-galactosidase A (αGal A, OMIM 301500)[1], [2] resulting in lysosomal accumulation of globotriaosylceramide (GL-3) in endothelial cells and other cell types in the body. The clinical spectrum of Fabry disease is remarkably heterogeneous, even within affected families [3]. Complications are mostly of vascular origin and comprise of progressive renal insufficiency, cardiac hypertrophy, arrhythmias and cerebral infarctions [4]. During childhood the main symptoms consist of episodes of excruciating pain in hands and feet, so-called acroparesthesias, and absence of sweating. Recently it has become clear that female carriers can also exhibit complications, although the disease usually has a more attenuated course in these patients [5].

In 2001 the European Medical Evaluation Agency (EMEA) approved registration of two recombinant enzyme preparations for the treatment of Fabry disease patients in Europe. Agalsidase alfa (Replagal™, Shire), produced by utilizing cultured human skin fibroblasts, is registered for use at a dose of 0.2 mg/kg biweekly, and agalsidase beta (Fabrazyme™, Genzyme), produced by expression of the αGal A gene in Chinese hamster ovary (CHO) cells, is registered for use at a dose of 1.0 mg/kg biweekly. The annual costs of therapy are almost equal for both preparations at the registered dose (around € 210.000 for a 70 kg patient), and as such agalsidase alfa is five times more expensive per milligram protein than agalsidase beta. Both products have shown their effectiveness in reducing GL-3 in tissue biopsies [6], [7], have favorable effect on renal function [8], [9] and reduce cardiac mass in patients with cardiac hypertrophy [8], [10]. A direct comparison of the two products in a clinical study has so far not been performed. Data from our laboratory, showed that both agalsidases had equal properties with respect to amino acid composition, specific activity, stability, and uptake by cultured fibroblasts [11]. More recent studies confirmed these findings [12], [13] except for minor differences in glycosylation [12] and mannose-6-phosphate receptor mediated cellular uptake [13]. These results should nevertheless be confirmed in a clinical study. All the more, since in contrast to these laboratory data, the early clinical studies on Fabry patients suggested that major clinical differences between the two enzyme preparations might exist. A more prominent effect on pain and renal function was observed using agalsidase alfa [7],[14] as compared to agalsidase beta [6]. Later studies suggested that agalsidase alfa treatment [8] gave a greater reduction in cardiac mass than agalsidase beta treatment [10]. However, the above-mentioned studies differed in patient inclusion criteria, outcome parameters and infused dose [15]. Definite conclusions on differences in clinical efficacy between the two agalsidase preparations can therefore not be drawn. The question that we wanted to answer was whether agalsidase alfa was clinically superior to agalsidase beta. Given the situation that the two enzyme preparations on the one hand exhibited identical biochemical properties, but on the other hand apparently showed differences in clinical outcome, this question would be best addressed by comparing the enzymes at equal dose. The choice for comparing 0.2 mg/kg per infusion instead of 1.0 mg/kg was a pragmatic one: the Dutch Government reimbursed the study, including the medication, and doses higher than the registered dose were not reimbursed. Thus, it was decided to compare the efficacy of and tolerability towards agalsidase alfa and beta in a prospective open label study in patients with symptomatic Fabry disease, who were randomly assigned to receive either agalsidase alfa or beta at an equal dose of 0.2 mg/kg biweekly. The outcome of this investigation is here described.

## Methods

The protocol for this trial and supporting CONSORT checklist are available as supporting information; see Checklist S1 and Trial Protocols S1 and S2.

### Participants

In the Netherlands, the Academic Medical Center (AMC) has been appointed by the Dutch government as the single national referral center for the treatment of Fabry patients, financially supported by a governmental grant through the Dutch Health Care Insurance Board (CVZ). The Center for Medical Genetics and Molecular Medicine, Haukeland University Hospital (HUH), Bergen, is a regional referral center for patients with Fabry disease in western Norway. All patients that were referred to the centers between May 2002 and December 2004 were asked to participate in the study. Eligible patients had a confirmed diagnosis of Fabry disease either by demonstration of reduction of αGal A activity in leukocytes (males) or DNA mutation analysis (females), were at least 18 years old and had to fulfill criteria for initiation of enzyme replacement therapy (table 1). Patients were excluded if they were undergoing dialysis, if they had a renal transplant, or if they were pregnant or lactating. All patients gave written informed consent. The study was approved by the Medical Ethical Committee (METC) and by the Dutch Central Committee on Research Involving Human Subjects (CCMO). This study is registered with the Dutch Trial Register and as an International Standard Randomised Clinical Trial, number ISRCTN45178534.

**Table 1:** Criteria for the initiation of enzyme replacement therapy for Fabry disease in the Netherlands.

Major criteria	1. Severe acroparesthesias that cannot be controlled satisfactorily with carbamazepine
	2. Decreased glomerular filtration rate<80 ml/min
	3. Proteinuria>300 mg/24 h
	4. Documented cerebrovascular accident
	5. Cardiac infarction
	6. Hypertrophic nonobstructive cardiomyopathy resulting in decreased exercise tolerance
	7. Rhythm disturbances necessitating a pacemaker
	8. Multiple lacunar infarctions on magnetic resonance imaging
Minor criteria	1. Documented transient ischemic attack
	2. Cardiac hypertrophy on echocardiography or magnetic resonance imaging
	3. Atrial fibrillation
	4. Intraventricular conduction abnormality
	5. Sensory hearing loss as shown on a hearing test
	6. Severe vertigo
	7. Microalbuminuria >50 mg/24 h
	8. Mild to moderate acroparesthesias
	9. Gastrointestinal complaints that cannot be explained by medical conditions other than Fabry disease

Eligible patients had a confirmed diagnosis of Fabry disease either by demonstration of reduction of αGal A activity in leukocytes (males) or DNA mutation analysis (females), were at least 18 years old and had to fulfill at least one major or two minor objective criteria.

### Interventions and Randomization

Patients who gave consent were randomly assigned to receive either agalsidase alfa or agalsidase beta at a dose of 0.2 mg/kg biweekly for a minimum of 12 months in an open label setting. Blinding of the enzyme products was not feasible, since the quality and storage life after rebottling could not be guaranteed. We set up a blinded permuted block randomization with a block-size of four. Severely affected (i.e. patients with glomerular filtration rate (GFR) <40 ml/min, cerebrovascular accident (CVA), restrictive cardiomyopathy and/or myocardial infarction) and less severely affected patients were randomized separately (i.e. a different set of randomization blocks was applied in both groups) in order to obtain an equal distribution of disease characteristics. Each block consisted of four envelopes that contained a paper that stated agalsidase alfa or beta. The envelopes were numbered consecutively (randomization number 1 through 4, 5 through 8, etc). These envelopes were generated and checked by 2 persons, who were not involved in obtaining informed consent. After informed consent was obtained, the patient was given an (consecutive) enrolment number (first patient was number 1 and so on). The envelop with the corresponding randomization number was opened by the investigator in presence of the patient. All patients subsequently received the allocated treatment.

In case of treatment failure (see criteria below) patients were advised to switch to agalsidase beta 1.0 mg/kg biweekly. Switching to a higher dose of agalsidase alfa was not allowed, since the additional costs were not covered. As part of standard clinical practice patients with microalbuminuria or proteinuria (>30 mg/24 h) received an angiotensin-converting enzyme inhibitor, and acetylsalicylic acid was prescribed in case of cerebral ischemic lesions and/or a history of transient ischemic attack (TIA).

### Objectives

The objective of the current study was to compare the efficacy of agalsidase alfa and beta at an equal dose of 0.2 mg/kg given once every two weeks, in Fabry patients with respect to 1. decrease in cardiac mass 2. changes in GFR, pain and GL-3 in serum and urine 3. number of treatment failures.

### Study design and sample size calculation

Prior to the start of our study, the available data from the first clinical investigation with agalsidase alfa [7], [14] suggested an improvement in renal function following enzyme treatment. The placebo-to-active-treatment-cross-over group showed an increase in GFR of 9 ml/min (≈10%) after 12 to 18 months of active treatment, whereas in the first 6 months of the study (placebo phase) these patients had shown a decline of 20 ml/min. In contrast, no improvement in renal function was reported in the randomized placebo controlled trial with agalsidase beta [6]. At that time point it was therefore fair to assume that at equal doses (0.2 mg/kg), agalsidase alfa treatment should have a better effect on kidney function than agalsidase beta treatment. For our analysis to have a power of 90% and an alpha-value of 5% (one-sided), it was predicted that 42 patients (21 in each group) were required to detect a difference in renal function improvement between agalsidase alfa and agalsidase beta treatment. However, during the course of the study subsequent literature reports indicated that treatment of Fabry patients with agalsidase alfa also stabilized rather than improved renal function (FDA briefing document[16], FOS outcome data [8]). This became clear after the inclusion of 29 patients. After ample considerations, we decided to change the primary endpoint in our study from improved renal function to reduction in left ventricular (LV) mass. This alteration of primary endpoint was made blinded; i.e. we were unaware of the effect of treatment on any outcome at that time. The choice for LVmass reduction as primary endpoint was based on literature reports indicating that agalsidase alfa treatment might cause a greater reduction in LVmass than agalsidase beta treatment. Using echocardiography, Beck et al. found an average 20% reduction of LVmass in Fabry patients with cardiac hypertrophy after 12 months of treatment with agalsidase alfa [8], while Weidemann et al. found a 10% reduction of LVmass after 1.0 mg/kg of agalsidase beta treatment as assessed by MRI [10]. The standard deviation in reduction in cardiac mass was assessed at 6.5% [16]. Accordingly, we assumed that at an equal dose of 0.2 mg/kg biweekly the reduction in LVmass would be more pronounced on agalsidase alfa than agalsidase beta treatment. Using a power of 90% and an alpha of 5% (one-sided), it was predicted that at least 18 patients (9 in each group) with an increased LVmass were required to detect a difference between the two enzyme treatments, i.e. a 10% larger reduction in LVmass by agalsidase alfa treatment than agalsidase beta treatment. Therefore, the inclusion was extended to have at least 9 patients with cardiac hypertrophy and complete echocardiography data in each group. Since in some patients follow-up cardiac data were missing, we assumed that at least 4 patients in each group might withdraw or had incomplete data, we decided to include 26 patients with cardiac hypertrophy. The additionally included patients were not selected at baseline for cardiac hypertrophy or any other parameter other than the pre-defined inclusion criteria.

As a separate analysis, we assessed the time to occurrence of treatment failure in both groups. Secondary endpoints in our study were GFR, GL-3 concentrations in urine and plasma, and pain score (BPI-3).

### Assessments of outcomes

Medical history, physical examination, routine chemical analysis and complete blood count, plasma for biochemical assessments, electrocardiography and 24-hour urinary samples and sediments were collected at baseline and every 3 months during treatment.

### Clinical and biochemical parameters

Left ventricular hypertrophy was assessed by ultrasound (General Electric Vivid 7) as earlier described (LVmass >259 g in males and >166 g in females [17]). Echocardiographic assessment of LVmass has shown to be highly reliable with an intraclass correlation coefficient (rho), an estimator of variability between replicate measurements, of 0.93. In addition, reproducibility has shown to be very high with a variation in mean LVmass of 0–2.6%, with some effect of regression to the mean in patients with severe cardiac hypertrophy [18]. Glomerular filtration rate (GFR) was measured at baseline and after 12 and 24 months of treatment by iothalamate/hippuran infusions [19](at the AMC) or iohexol infusions [20](at the HUH). Both tests have shown to be very accurate with an intratest coefficient of variation of 1.93% and 5.59%, respectively. In a few instances cardiac ultrasound and GFR measurements were not performed within the required timeframe (±2 months of planned assessment) for logistical reasons. This happened in 5 cases for both measurements at baseline, and 9 and 5 cases at 12 months for cardiac ultrasound and GFR, respectively. Pain was assessed through the Brief Pain Inventory (BPI) as earlier described [7] at baseline and after 12 and 24 months and was only obtained from the AMC patients. Brain MRI was assessed at baseline and at 12 and 24 months for the absence or presence of lacunar infarctions (score 0 or 1 respectively), where a score of 2 represents a new (lacunar) infarction (see table 2). Specialists who evaluated all ECGs, echocardigrams, GFR measurements and MRIs, were blinded for the assignment of patients to treatment groups and were not informed of previous results at the time of testing. Overall severity of disease was assessed using the Mainz Severity Score Index (MSSI) [21]

**Table 2:** Patient clinical data at baseline and after 12 and 24 months of treatment.

					GFR (ml/min)			LVmass (g)			MRI			GL-3 plasma (umol/l)			GL-3 urine (nmol/24 h)		
Pt	Sex	Prep	Age	Ab	0	12	24	0	12	24	0	12	24	0	12	24	0	12	24
1	f	beta	52	0	80	105	81	251	.	212	1	1	1	3,17	2,68	2,55	1247	775	460
2	f	beta	47	0	117	111		213	297	205	1	1	1	4,18	3,01		246	155	
3	f	beta	43	0	108	102	110	369	317	333	1	1	1	1,46	1,4	2,09	416	514	482
4	f	beta	47	0	121	114	110	.	358	401	1	1	1	0,77	1,43	1,34	441	176	248
5	f	beta	71	0	75	82		329	174		1	1		2,34	2,48		345	256	
6^a^	f	beta	54		72	89	69	260	219	196	.	.		.	.		.	.	
7^a^	f	beta	76		70	66	55	517	331		.	.		.	.		.	.	
8	m	beta	35	0	106	115	104	270	401	401	0	0	0	3,31	1,89	1,74	2100	884	ns
9	m	beta	61	1	53	56	49	353	.	471	1	.	2	9,1	3,93	4,17	2860	1720	2415
10	m	beta	47	1	.	.		316	337	283	1	.	2	3,06	1,97	1,79	2464	2160	1173
11	m	beta	50	0	126	111	109	260	177	217	1	2		1,39	1,73	0,92	602	97	123
12	m	beta	45	1	.	.		336	296		1	1	1	6,47	3,66	4,3	3724	4524	2856
13	m	beta	25	1	154	159		192	169		1	1		4,1	1,85		802	557	
14	m	beta	50	1	.	.		233	233	356	1	1	1	5,71	3,27	3,44	760	2210	3870
15	m	beta	45	1	149	159	144	313	.	334	0	0	0	3,71	3,48	2,37	2111	3306	2569
16^a^	m	beta	24		113	123	125	479	253	271	.	.		.	.		.	.	
**Median**			**47**		**108**	**111**	**107**	**313**	**296**	**308**				**3,31**	**2,48**	**2,23**	**802**	**775**	**1173**
**(sd)**			**(14)**		**(31)**	**(30)**	**(31)**	**(91)**	**(76)**	**(90)**				**(2,3)**	**(0,9)**	**(1,1)**	**(1133)**	**(1382)**	**(1372)**
**(range)**			**(24–76)**		**(53–154)**	**(56–159)**	**(49–144)**	**(192–517)**	**(169–401)**	**(196–471)**				**(0,77–9,1)**	**(1,40–3,93)**	**(0,92–4,3)**	**(246–3724)**	**(97–4524)**	**(123–3870)**
17	f	alfa	60	0	73	63		479	414		1	1		2,82	2,44		657	431	
18	f	alfa	43	0	109	118	118	.	321	292	0	0	0	2,61	2,1	2,45	792	453	325
19	f	alfa	60	0	61	64	65	200	171	231	1	1	1	3,28	3,6	3,22	884	195	117
20^a^	f	alfa	45		86	92		177	180		.	.		.	.		.	.	
21	f	alfa	50	0	101	114	110	270	303	259	1	1	2	2,93	2,02	2,7	520	292	417
22	f	alfa	47	0	86	66	67	331	270	297	1	1	1	3,22	3,07	2,53	785	337	378
23	f	alfa	35	0	122	112		221	197		0	0		2,45	2,13		802	708	
24	f	alfa	35	0	105	77		180	157		1	1		2,67	2,57		996	464	
25	f	alfa	58	0	30	22		195	235		1	1		2,14	1,45		ns	ns	
26	m	alfa	34	1	100	124	83	190	.	310	0	0	0	6,91	4,2	6,3	2016	2142	2803
27	m	alfa	55	1	.	.		404	.		1	.		6,06	3,74		.	.	
28	m	alfa	22	0	124	145	129	.	.		0	0	0	7,46	3,07	3	2233	349	713
29	m	alfa	20	0	132	150	132	.	.		0	0	0	5,95	3,55	2,7	1254	1465	546
30	m	alfa	19	0	136	147	158	.	220	252	0	0	0	7,03	3,53	3,06	1248	487	ns
31	m	alfa	47	0	22	16	14	288	424	396	1	1	1	9,74	3,93	1,89	5681	742	153
32	m	alfa	55	1	.	.		377	.	502	1	1	1	8,38	6,42	7,85	3472	3400	3500
33	m	alfa	42	1	98	133	100	317	.	296	1	1	2	8,8	4,96	7,19	1702	3052	1551
34^a^	m	alfa	31		86	88	83	420	253	196	.	.		.	.		.	.	
**Median**			**44**		**99**	**102**	**100**	**279**	**244**	**294**				**4,6**	**3,3**	**3**	**1122**	**476**	**482**
**(sd)**			**(13)**		**(33)**	**(42)**	**(40)**	**(101)**	**(89)**	**(87)**				**(2,6)**	**(1,2)**	**(2,1)**	**(1409)**	**(1069)**	**(1190)**
**(range)**			**(19–60)**		**(22–136)**	**(16–150)**	**(14–158)**	**(177–479)**	**(157–424)**	**(196–502)**				**(2,14–9,74)**	**(1,45–6,42)**	**(1,89–7,85)**	**(520–5681)**	**(195–3400)**	**(117–3500)**
**Δ groups (p)**			0.25		0.50			0.50			0.34			0.14			0.40		

Prep, preparation; f, female; m, male; alfa, agalsidase alfa; beta, agalsidase beta; Ab, antibodies; 0, normal; 1, abnormal; 2, new lesion; ^a^, patients included in Norway; ns, no sediment obtained after spinning of urine; Δ, difference between two treatment groups.

Concentrations of GL-3 in urine was measured as described before [22], [23]. Levels of GL-3 in plasma were measured by high pressure liquid chromatography (HPLC) (Groener et.al., submitted manuscript). Lipids were extracted from 50 µl plasma according to Folch [24]. Glycosphingolipids were deacylated by a modification of the microwave-assisted hydrolysis, as described by Taketomi et al. [25]. The produced lysoglycosphingolipids were derivatized with O-phtaladelhyde, separated on a reverse phase HPLC column and measured with a fluorescence detector. GL-3 levels were measured at baseline and every 3 months during treatment. Plasma samples were also evaluated for the presence of antibodies towards the enzyme products by ELISA, as previously described [23]. A dilution of 1:8 was determined as the lower detection limit, which is applicable to both agalsidase alfa as well as beta. Lipid and antibody data were only collected for the Dutch cohort (29/34 patients).

### Definition of treatment failure

Treatment failure was defined as progression of renal disease (33% increase in serum creatinine, need for dialysis or transplantation), progression of cardiac disease (new infarction, need for cardioversion, or anti-arrythmic drugs, heart-failure necessitating hospitalization), or occurrence of a new CVA as diagnosed by a neurologist or new lacunar infarctions on magnetic resonance imaging (MRI) as assessed by an experienced neuroradiologist.

### Statistical methods

Efficacy of treatment was analyzed as a change in parameters between baseline and 12 and 24 months of treatment for every parameter studied. Results are expressed as median and range. Differences between the different treatment groups were calculated using the Mann Whitney U-test or chi-squared test. Differences between baseline and 12 months and baseline and 24 months of treatment were calculated by the Wilcoxon signed rank test. Correlations between variables are described by assessment of Spearman rank correlation coefficients. The percent of patients experiencing no treatment failure was studied by Kaplan-Meier analysis. The logrank test was used to compare time to treatment failure between the two groups. P values <0.05 were considered significant.

## Results

### Baseline characteristics

**Figure 1:**
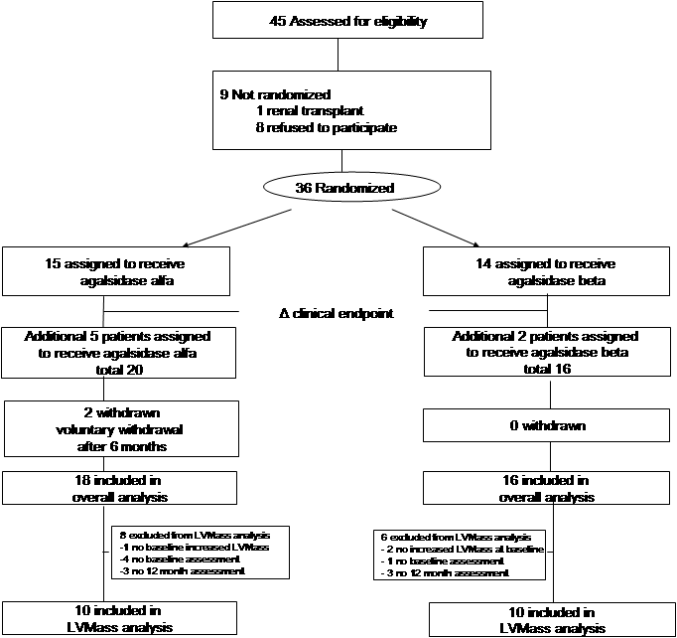
Study flow chart. Δ clinical endpoint: change of primary endpoint from improvement of renal function to reduction in left ventricular mass.

Forty-five patients were assessed for eligibility (figure 1). One male patient did not meet the inclusion criteria due to a renal transplant. Eight patients chose to be treated with agalsidase beta at 1.0 mg/kg, either because family members already received this treatment (3 cases), or for other unrelated reasons. These patients did not differ with respect to disease severity from the patients participating in the study. Thirty-six patients, 18 males and 18 females, were included in the study. Two mildly affected female patients withdrew at their own request after 6 months of agalsidase alfa therapy. Neither of these women had suffered from treatment failure during the 6 months study period. The thirty-four patients remaining had at least 12 months of follow up. A sub-group of patients (25/34) was followed for more than 24 months of treatment. The baseline characteristics were similar for both treatment groups (table 2).

Concomitant drug therapy at baseline included angiotensin-converting enzyme inhibitors or angiotensin-I blockers (8 patients in the agalsidase alfa and 4 in the agalsidase beta group), beta blockers (4 in the agalsidase alfa and 3 in the agalsidase beta group), diuretics (1 in the agalsidase alfa 3 in the agalsidase beta group ), calcium channel blockers (3 in the agalsidase beta group) and acetylsalicylic acid (11 in the agalsidase alfa and 11 in the agalsidase beta group). All co-medications were continued throughout the study. Changes in co-medication during the trial were minimal: in 3 patients (2 in the agalsidase alfa group and one in the agalsidase beta group) a dose change of angiotensin-converting enzyme inhibitor or angiotensin-I blocker was made, and in one patient (agalsidase alfa) an angiotensin-converting enzyme inhibitor was started during the course of the study. Four patients (3 in the agalsidase alfa and 1 in the agalsidase beta group) were treated for hypertension (untreated blood pressure >140/90 mmHg). All were well controlled.

### Effects of agalsidase alfa and beta therapies on clinical parameters

#### Primary endpoint: cardiac hypertrophy

Echocardiographic measurements were performed in 29 patients at baseline (14 in the agalsidase alfa and 15 in the agalsidase beta group). Twenty-six of these patients had an increased LVmass at baseline (13 in the agalsidase alfa and 13 in the agalsidase beta group). Of these patients follow up data were available from 20 patients at 12 months (10 in both treatment groups) and 17 patients at 24 months (7 agalsidase alfa, 10 agalsidase beta). There was no difference in median cardiac mass between the two groups at baseline (agalsidase alfa: 288 g, range 177 to 479, agalsidase beta: 316 g, range 213 to 517, p = 0.48). At 12 months, no significant reduction in cardiac mass was seen in both groups. Median change was −11% (−23 g, range −167 to 136 g, p = 0.51) following agalsidase alfa treatment and −15% (−46 g, range −226 to 131 g, p = 0.17) after 12 months of agalsidase beta treatment. These results were not different between the treatment groups (p = 0.3). The median change in the combined groups after 12 months of treatment was −11% (−35 g, range −226 to 136 g; p = 0.10), and this was also non-significant. Data available of patients who reached 24 months follow up gave a consistent pattern. Sub-analysis of the data showed that all patients with poor renal function (GFR<60 ml/min, n = 4, including patient 27 who had a baseline creatinine clearance (calculated from 24 h urine collection) of 41 ml/min) had an increase of LVmass during treatment. Without these patients a significant reduction in LVmass (14%, p = 0.03) after 12 months of treatment was observed for the combined groups, which did not reach significance for the two treatment groups separately (p = 0.09 and 0.17 for agalsidase alfa and beta, respectively).

#### Secondary endpoint: Glomerular filtration rate (GFR) and proteinuria

Median GFR was not different between the two treatment groups at baseline (agalsidase alfa: 99 ml/min, range 22 to 136, agalsidase beta: 108, range 53 to 154, p = 0.50). Following 12 months of treatment no significant change could be observed. A slight increase of 5 ml/min in both groups was noted (ranges: agalsidase alfa −28 to 35 p = 0.37, and agalsidase beta −15 to 25, p = 0.23). Two patients (patient 25 and 31, table 2), both presenting a low GFR at start of treatment, showed progression of renal disease during therapy as reflected by a >33% increased serum creatinine. In patients evaluated after 24 months of treatment (n = 21), no additional changes in renal function were observed. Exclusion of the patients with a baseline GFR <60 ml/min did not change the results.

Urinary protein data were available for the Dutch patients. There was no difference in baseline levels between the two treatment groups: agalsidase alfa: 0.25 g/24 h (range 0.06 to 2.65), agalsidase beta: 0.24 g/24 h (range 0.10 to 0.68), p = 0.59. The values did not change following 12 and 24 months of treatment (agalsidase alfa at 12 months: 0.30 g/24 h (range 0.08 to 2.83, p = 0.35) and at 24 months: 0.27 g/24 h (range 0.10 to 1.65, p = 0.70), agalsidase beta at 12 months: 0.20 g/24 h (range 0.08 to 0.50, p = 0.33) and at 24 months: 0.15 g/24 h (range 0.06 to 0.57, p = 0.28)). These changes in proteinuria were not different between the two treatment groups (p = 0.16 and p = 0.33, for 12 and 24 months respectively). A change in dose of angiotensin-converting enzyme inhibitors or angiotensin-I blockers did not influence the results.

#### Secondary endpoint: Pain Score

Data on pain score were available for 15 patients in the agalsidase alfa group and for 13 patients in the agalsidase beta group. No pain scores were available from the patients included at the HUH and one patient from the AMC. Since the Norwegian patients were not different with respect to disease severity and gender (three females and two males), we expected no difference in outcome. At baseline, pain scores were not different (agalsidase alfa: median 4, range 0 to 8, agalsidase beta: median 4, range 0 -7).

No significant reduction of pain score (BPI-3) after 12 months of treatment was noted in any of the groups: alfa 0 (range −5 to 1), beta −1.5 (range −4 to 3). Evaluation after 24 months of treatment (n = 20) did not change this result.

#### Secondary endpoint: Antibodies and biochemical parameters (GL-3 in urine and plasma)

Anti-agalsidase antibodies and GL-3 levels in urine and plasma were only analyzed in the Dutch Fabry patients. Antibodies were found in 10/16 males (4/8 agalsidase alfa, 6/8 agalsidase beta, χ^2^ = 1.07, p = 0.3); no female patient developed antibodies. At 6 months, titer levels ranged from 1/64 to 1/32768 in agalsidase alfa treated males and from 1/256 to 1/16384 in agalsidase beta treated males. After 12 months of treatment only two males (agalsidase beta) had a decline of antibody titer. Infusion related side effects were seen in 3/10 antibody positive male patients, 1/4 treated with agalsidase alfa and 2/6 treated with agalsidase beta. These side effects were chills and fever, followed by acroparesthesias.

All but two females in the agalsidase beta group had elevated urinary GL-3 levels (>400 nmol/24 h) at baseline. In the patients with elevated baseline levels a median decrease of 284 nmol/24 h (range −4939 to 1350, p = 0.04) in the agalsidase alfa group and 265 nmol/24 hr (range −1216 to 1450, p = 0.66) in the agalsidase beta group was found after 12 months of treatment. This reduction in GL-3 was not significantly different between the two groups (p = 0.65). As antibodies may interfere with GL-3 clearance we performed an exploratory subgroup analysis. This revealed a major difference in urinary GL-3 reduction upon therapy between male patients with and without antibodies. In all antibody positive males, urinary GL-3 levels failed to decline after 12 months of treatment (126 nmol/24 h, range −1140 to 1450, p = 0.31 (n = 10)) whereas a reduction could be detected in males without antibodies (−989 nmol/24 h, range −4939 to 216, p = 0.04 (n = 6)). This difference in urinary GL-3 reduction between males with and without antibodies was statistically significant (p = 0.02), and a similar trend was also observed in male patients (n = 12) analyzed after 24 months of treatment.

Plasma GL-3 was elevated at baseline (>3.18 umol/l) in 10/16 agalsidase alfa (2 females) and 8/13 agalsidase beta (1 female) treated patients. In those patients who showed elevated GL-3 levels at baseline, a decrease of 2.56 (range −5.81 to 0.32, p = 0.009) after 12 months of agalsidase alfa treatment was seen. Similar results were seen in the agalsidase beta treated patients with a reduction of 1.84 (range −5.17 to −0.23, p = 0.012). This reduction of GL-3 was not different between the two treatment groups (p = 0.46). Twenty-four months follow up data (n = 15) showed a similar pattern. Antibodies resulted in a less robust decrease of plasma GL-3 levels in male patients after 12 months of treatment (ab+ −2.44 umol/l, range −5.17 to −0.23, p = 0.005 (n = 10); ab- −3.5 umol/l, range −5.81 to −1.42, p = 0.04 (n = 6)). Again a similar trend was noted in the male patients (n = 13) analyzed after 24 months of treatment.

### Treatment failure

**Figure 2:**
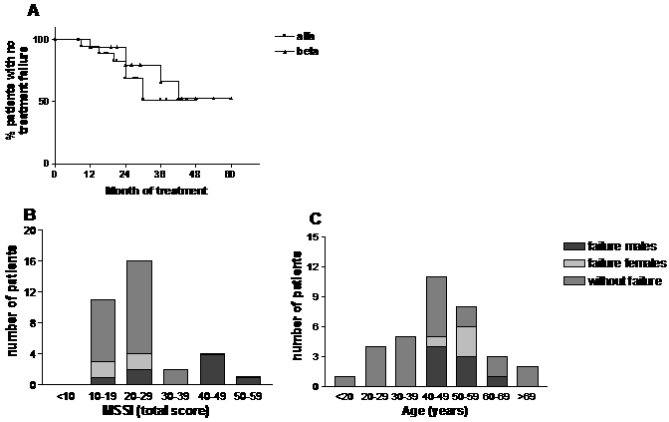
A: Kaplan Meier survival analysis of percent patients with no treatment failure in both treatment groups. B: Number of patients with treatment failure displayed per 10 MSSI points. C: Number of patients with treatment failure displayed per age decade.

Treatment failure within 24 months of treatment was seen in 6 male patients (3 in each treatment group) and 2 female patients (both agalsidase alfa). The time to the occurrence of treatment failure did not differ between the two treatment groups; χ^2^ = 0.38 p = 0.54 (figure 2A). Additional analysis including the two female patients that withdrew volunatrily, assuming they had been ‘event-free’ for 24 months, does not result in a different conclusion (χ^2^ = 0.18, p = 0.66). The three male patients (patients 9,10 and 11, table 2) with treatment failure on agalsidase beta showed progression of MRI abnormalities. This was also observed in one male (patient 33) and one female patient (patient 21) in the agalsidase alfa group. Renal insufficiency (increase of serum creatinine >33%) progressed in one male (patient 31) and one female patient (patient 25) treated with agalsidase alfa, with baseline GFR of 22 and 30 ml/min, respectively. The increase in serum creatinine corresponded with a decrease in GFR of 27.2% and 26.8%, respectively (see table 2). One male patient (patient 27) died of multiple cerebral infarctions after 20 months of agalsidase alfa treatment. Data analysis of patients receiving ERT for more than 24 months showed treatment failure in four additional patients (2 in each treatment group). One male patient (patient 12) and one female patient (patient 1) suffered from atrial fibrillation after 42 and 36 months of agalsidase beta treatment, respectively. Atrial fibrillation also occurred in a male patient (patient 32) treated with agalsidase alfa for 30 months. Progression of renal insufficiency was seen in a female patient (patient 22) following 30 months of agalsidase alfa treatment. All patients showing treatment failure were switched to infusion with agalsidase beta at standard dose (1.0 mg/kg biweekly). Even after this switch, further progression of disease was seen in 5 patients (2 initially on agalsidase alfa and 3 initially on agalsidase beta, all progression of MRI abnormalities). Pre-treatment disease severity as assessed by the MSSI score was associated with the occurrence of treatment failure in males (figure 2B): average MSSI was 44 (range 17–59) in male patients with treatment failure and 21 (range 12–37) in those without treatment failure (p = 0.02). Age was also associated with treatment failure; all patients with progression were significantly older than the patients without treatment failure (p = 0.02), all being above 40 years of age (figure 2C, median all patients 50 (42–61), females 51 (47–58), males 49 (42–61)).

The induction of antibodies did not correlate with the occurrence of treatment failure: 6/10 male patients with antibodies and 2/6 without antibodies developed treatment failure. Further exploratory analysis was performed on the relation between GL-3 levels and treatment failure. When urinary GL-3 levels of patients who suffered from treatment failure were compared with those who did not, no differences in baseline levels and extent of decrease were shown. However, a trend towards higher baseline urinary GL-3 levels was seen in patients with treatment failure (2083 nmol/24 h, range 520 to 5681 (n = 10), as compared to 802 nmol/24 h, range 246 to 2233 (n = 17), p = 0.06). Reduction in urinary GL-3 was 19% and 22% after 12 and 24 months in patients with, and 28% and 42% in patients without treatment failure (p = 0.60 and p = 0.39, respectively). Plasma GL-3 levels of patients who suffered from treatment failure were not different from the levels found in patients who did not: average baseline levels of plasma GL-3 in treatment failures were 4.64 umol/l (range 1.39 to 9.74 (n = 12)) and non-treatment failures 3.31 umol/l (range 0.77 to 7.46 (n = 17)) (p = 0.28). Reductions in GL-3 were 30% and 22% in treatment failures versus 15% and 39% in non-treatment failures, respectively, at 12 (p = 0.25) and 24 months (p = 0.61).

### Adverse events

Serious adverse events, which did not fulfill the criteria for treatment failure, resulting in hospitalization or initiation of medication were seen in one agalsidase alfa and three agalsidase beta treated patients. One male patient (patient 32) treated with agalsidase alfa presented with intermittent claudication of the right leg due to vascular stenosis after 15 months of therapy, which was treated with lifestyle advice. During agalsidase beta treatment one female patient (patient 1) reported acute hearing loss after 18 months, one male (patient 9) presented with severe vertigo after 12 months and one male (patient 11) showed progressive sensomotor polyneuropathy and oesophagitis after 12 months of treatment. These last two events were considered not to be related to Fabry disease. As reported above, only three antibody positive male patients experienced infusion related chills and fever, 1 treated with agalsidase alfa and 2 treated with agalsidase beta. The incidence of mild to moderate adverse events (grade 1 or 2 nausea, dizziness, headache and diarrhea) was low and not different between the two treatment groups.

## Discussion

Since ERT became available for Fabry disease, it has been extensively debated which of the two available enzyme preparations is most potent. The only direct comparative studies that have been performed are either *in vitro* studies or studies in mice, showing no important differences. [12], [13], [15], [26]–[29]. Studies in Fabry patients have so far focused on the clinical and biochemical effects of both products separately. All studies with agalsidase alfa were performed with a dose of 0.2 mg/kg every two weeks. The first placebo-controlled clinical trial with agalsidase alfa has shown a beneficial effect on pain. In that study, and the EMEA report, stable renal function in the agalsidase group and improvement in creatinine clearance after cross over from placebo to active treatment was reported, as well as improved renal histology and decline a in GL-3 levels in the urine [7], [14]. Subsequently, in an open study, improvement of sweat function and warm or cold sensation in feet was reported [30]. More recently, renal outcome was described following 4–4.5 years of agalsidase alfa therapy, showing stabilization in patients with preserved renal function, but gradual deterioration in advanced kidney disease [31]. The Fabry Outcome Survey, a large database including mainly data on treated patients, created for post-marketing surveillance, reported stabilisation of renal function as well as decreases in cardiac mass, improvement of pain and quality of life following 12 to 24 months of agalsidase alfa treatment [8], [32], [33]. All clinical studies involving agalsidase beta were performed at 1.0 mg/kg every two weeks. The first placebo controlled trial with agalsidase beta focused mainly on surrogate endpoints, including clearance of GL-3 from renal endothelium as well as a reduction in GL-3 accumulation in the heart, skin and plasma [6]. After 30 to 36 months of open label treatment, renal function had stabilized in most, but not all, patients and a persistent decrease in plasma GL-3 and a further clearance of accumulation from the skin was reported [9]. Two open label studies reported decrease of LVmass and an improvement in myocardial function following 12 months of treatment [10] [34]. Recently Breunig et al. [35] recorded in an open label study in 26 patients that clinical endpoints such as death, cardiac and cerebrovascular events and renal failure occurred 12 times in 9 patients, all with advanced renal insufficiency. The very recently reported results of a phase IV placebo-controlled study, in which the time to similar clinical events was analyzed in 82 patients with mild to moderate kidney disease, revealed that treatment resulted in less complications, but also that this beneficial effect was preferentially seen in patients with less severe kidney disease [36]. In summary, the early studies reported mainly a beneficial effect on clinical outcome (pain, renal function) with agalsidase alfa, and reductions in stored GL-3 in the agalsidase beta studies. The following studies showed less effect on renal function, but consistent reductions in cardiac mass, with especially promising improvements using agalsidase alfa. In the most recent studies, however, there is growing evidence that advanced disease, especially kidney disease, negatively influences the outcome. Although agalsidase alfa was believed to exhibit the most positive effects on clinical endpoints, a head-to-head comparative study was needed to address whether indeed there was superiority of agalsidase alfa. Therefore, we have studied the effectiveness of treatment with agalsidase alfa and agalsidase beta at equal doses (0.2 mg/kg biweekly) in Fabry disease patients in a randomized open label study. Our primary endpoint was based upon previous reports showing a reduction in LVmass upon treatment [8], [10]. The response on cardiac hypertrophy was less than expected from these studies with patients showing an increase as well as a decrease in LVmass, resulting in an overall reduction that was not statistically significant. The changes in LVmass did not differ between the two treatment groups, neither after analyzing treatment effects in male and female patients separately. A closer look at the effects of ERT on LVmass suggested that the trend towards a decrease was slightly more prominent in the agalsidase beta group than in the agalsidase alfa group (15% vs. 11% after 12 months and 11% vs. 5% after 24 months), but this difference was not statistically significant. The question remains whether this variable effect can also be attributed to pretreatment severity of disease and age as compared to the reports in the literature. In the study by Weidemann et al.[10], a 10% decrease in LVmass as measured by MRI and following 12 months of treatment with a standard dose of agalsidase beta (1.0 mg/kg biweekly) was observed. When comparing the age of this group with our cohort, our cohort appears to be slightly older (47 years vs. 42 years), which may negatively influence therapeutic effects. In addition, mean left ventricular mass at baseline was lower (201 g) than in our group (316 g). However, MRI may give different LVmass measurements than echocardiography, and it is therefore difficult to compare the baseline degree of hypertrophy in both cohorts. It is also possible that a five times higher dose used in the Weidemann et al. study resulted in a more consistent beneficial response in this group. As mentioned above, the same group described that in patients with severe renal impairment, cardiac hypertrophy was more extensively present and even progressed upon therapy [35]. Similarly, in our cohort the patients with a GFR <60 ml/min were the ones that had a further increase in cardiac mass despite ERT. Re-analysis of the cardiac data, without these patients and after combining both treatment groups, indeed revealed a significant reduction of LVmass of 14% after 12 months of treatment. This underscores that severe renal impairment in Fabry disease is associated with irreversible cardiac hypertrophy [35]. Beck et al.[8] reported a 20% reduction in LVmass following 12 months of standard dose agalsidase alfa treatment (0.2 mg/kg biweekly). This is more prominent than the response observed in our agalsidase alfa group. Mean LVmass at baseline was higher than in our agalsidase alfa treatment cohort (70 g/m^2.7^ = 300 g for a 1.70 m individual, versus 221 g in our agalsidase alfa group), which may indicate more advanced disease. Although slight differences may exist for the sub-group in which cardiac evaluation is performed in the Beck et al study, the baseline GFR and age as presented in this paper was not different from our group.

In line with the similarity in clinical responses to agalsidase alfa and beta treatments at 0.2 mg/kg biweekly, the reductions in plasma and urinary GL-3 in both treatment groups were not significantly different. We noted that reduction of urinary GL-3 tended to be better in male Fabry patients treated with agalsidase alfa than agalsidase beta. The higher number of male patients developing antibodies towards agalsidase beta (6/8) as compared to agalsidase alfa therapy (4/8) may explain this. We and others have reported earlier that antibody formation interferes with GL-3 clearance [23], [37]. Also in the current study, an association between the emergence of antibodies and absence of decline in urinary GL-3 was established. Notably, although the occurrence of antibodies correlated with less reduction of GL-3 levels in Fabry patients, this was not associated with significantly poorer responses in key clinical endpoints such as cardiac hypertrophy, renal function and brain complications. Previous studies by Eng et al.[6] and Schiffmann et al.[38] reported emergence of antibodies in 88% (51/58) agalsidase beta 1.0 mg/kg treated and 56% (14/25) agalsidase alfa 0.2 mg/kg treated male patients. These results are difficult to compare since different enzyme dosages and different methods for antibody detection were employed in the studies. The antibody results from our study need to be interpreted with great caution as we only performed analysis in a small group of patients. It is, however, possible that agalsidase alfa is intrinsically less antigenic than agalsidase beta in male Fabry patients.

One of our most important observations is that the efficacy of ERT, whether using agalsidase alfa or beta, in general is disappointing. Both groups had a high rate of treatment failure, approaching 25% of patients within the 24 months study period, and increasing to about a third of the patients when patients who received treatment for more than 24 months were also included (12/34 patients). The incidence of treatment failure was not different between the agalsidase alfa and beta treatment groups (7/18 patients on agalsidase alfa and 5/16 patients on agalsidase beta). Increase in cardiac mass was unexpected to occur, and as such was not included in the treatment failure criteria. In retrospect, 7 patients experienced an increment in LVmass, 4 in the agalsidase alfa and 3 in the agalsidase beta group at 12 months. A further analysis of the patients with disease progression on ERT clearly showed that treatment failure occurred predominantly in patients with more extensive pre-treatment manifestations, as evidenced by a higher MSSI score. Consistent with this, the patients with treatment failures were generally older. Before the age of 40 and with an MSSI score <17, no failures occurred. In addition, all patients with a GFR <60 ml/min had progression of disease, including two patients who had a further decline in renal function. This is in line with the previously discussed more recently published studies on long term treatment in patients with advanced kidney disease, in whom further deterioration of renal insufficiency during ERT was observed [9], [31], [35].

Since the overall treatment effects were less prominent than anticipated, the comparison of the efficacy of agalsidase alfa versus agalsidase beta at equal dose has become difficult. When considering the observed reductions in cardiac mass, including the larger variability in these reductions than expected, it becomes apparent that the number of patients in our study has been too small to provide a definite answer to the question whether these products have different effects at the same dose. Despite this limitation, it remains clear that in both groups, with comparable baseline disease parameters, the number of failures to treatment was not different. It is uncertain whether a higher dose of agalsidase alfa or beta would have resulted in better responses (less treatment failures) since the damage in this severely affected older patient group will probably not respond to any therapy. This is supported by the observation that further disease progression is seen in a considerable subset of these patients after switching to 1.0 mg/kg of agalsidase beta. In this group with advanced disease, enzyme therapy may still have some effect as it has been suggested that the slope of decline in renal function may be decreased [35], [38].

When we compare the outcome in the non-treatment failures, no difference in response with respect to cardiac mass and GFR was found. It is possible that a minor difference in effect between the two enzymes will be detected if a much larger group of patients would be studied. However, it is very unlikely that these trials will be performed given the rarity of the disorder as well as the fact that it will be almost impossible to prospectively collect large numbers of unbiased data in a post-marketing setting.

As has also been suggested by others, early treatment may be the best option for Fabry disease patients to profit from ERT [8], [9], [35], [39]. Since treatment is very expensive, not without side effects, and only possible through frequent intravenous infusions that can carry a high burden on (young) patients, it is now a challenge to find the threshold where therapy is still capable of preventing or reversing organ damage caused by the disease. A long-term study in young, pre-symptomatic males who are randomized to either immediate therapy or later institution of ERT could eventually resolve this issue. International guidelines for these costly therapies should derive from such studies.

In conclusion, our study has revealed similar effects on clinical parameters (cardiac hypertrophy, glomerular filtration rate) and on plasma and urinary GL-3 reduction after 12 and 24 months of treatment with either agalsidase alfa or beta at a dose of 0.2 mg/kg biweekly. Although the number of patients in this study is small, it is unlikely that large differences in clinical potency exist between the two enzymes at equal dose. Treatment failure occurred relatively often in both groups, and further progression of disease could not be prevented after a switch to 1.0 mg/kg agalsidase beta. Treatment failure seems related to age and severe pre-treatment disease, supporting the hypothesis that early initiation of ERT in the disease course may be the only way to prevent long term complications.

## Supporting Information

Trial Protocol S1 (0.26 MB PDF)

Trial Protocol S2 Trial Protocol (amended)
(0.27 MB PDF)

Checklist S1 CONSORT Checklist
(0.27 MB PDF)
