# Supplementary material for: Treatment of Fabry Disease: Outcome of a Comparative Trial with Agalsidase Alfa or Beta at a Dose of 0.2 mg/kg
Source: PLoS One. 2007 Jul 11;2(7):e598. doi: 10.1371/journal.pone.0000598 (PMC1913555; doi:10.1371/journal.pone.0000598)
Supplement: Trial Protocol S2 — Trial Protocol (amended) (0.27 MB PDF) [file pone.0000598.s002.pdf]

TREATMENT PROTOCOL

TREATMENT OF FABRY PATIENTS > 18 YEARS WITH ENZYME SUPPLEMENTATION THERAPY: COMPARISON OF EFFICACY AND TOXICITY OF LOW DOSE (0,2 MG/KG) FABRAZYME (AGALSIDASE BETA) OR REPLAGAL (AGALSIDASE ALFA).

Version 2, December 2004

Involved physicians and organizations:

Coordinating center:

Academic Medical Center. Amsterdam
Principal investigator: Carla E.M. Hollak, MD, PhD, heamatologist
Study coordinator: Anouk C. Vedder, MD
Gabor E. Linthorst, MD

Involved physicians and nurses:
Prof. Dr. H.S.A. Heymans, pediatrician
Dr. F.A. Wijburg, pediatrician
Prof. Dr. R.T. Krediet, nephrologist
Dr. F.J. Bemelman, nephrologist
Dr W Grolman, ENT-specialist
Dr LR Dekker, cardiologist
Dr. B.J. Bouma, cardiologist
Dr. C. Majoie, neuroradiologist
Prof.dr. J.M.F.G. Aerts, biochemist
Mrs. E.E. Ormel, study nurse
Mrs. M.G. Wiersma, study nurse
Drs. P.N.J. Langendijk, apotheker

Collaborative Dutch physicians:

Antonius ziekenhuis Nieuwegein:
Dr. P.H.TH. J. Slee, internist

Westfries Gasthuis, Hoorn:
Dr. L.J. van Oudheusden, pediatrician.

Academisch Ziekenhuis Groningen:
Dr. W.J. van Son, nephrologist

Involved organizations:

Dutch Clinical Genetic Centers of Groningen, Leiden, Nijmegen, Rotterdam and Utrecht.

Index

Involved physicians and organizations: 2
Index 3
Summary 4
INTRODUCTION 6
PROTOCOL OBJECTIVES 7
INVESTIGATIONAL PLAN 7
Study design and scheme 7
Duration of treatment protocol. 7
PATIENT POPULATION AND SELECTION 7
Criteria for treatment 7
Exclusion criteria 8
Patient withdrawal 8
TREATMENTS 8
Randomization 8
Administration of low-dose agalsidase (Fabrazyme or Replagal 0,2 mg/kg) 8
Prior and concomitant Medications 9
Investigational products 9
Fabrazyme 9
Replagal 10
Packaging 11
Reconstitution 11
Evaluation and monitoring of treatment: 11
Post-infusion α-Gal activity 11
Month 1,2 12
Month 3, and following 3 months 12
Month 6,12, 24 12
EFFICACY AND SAFETY VARIABLES 12
Evaluation of GFR 13
Evaluation of skin tissue 13
Pain-scores (BPI) and quality of life (SF-36) 13
Urinary Kreatinin and protein 13
Urinary Glycolipid 13
Plasma Glycolipid 13
Physical examination 13
Electrogradiogram 13
Echocardiogram 13
Safety lab assessments 13
Antibody testing 14
MRI assessments 14
Hearing test 14
Adverse and serious adverse experiences 14
Statistical methods and planned analyses 14
Efficacy endpoints: 14
Efficacy analysis 15
Sample size estimation to detect a possible difference between Replagal and Fabrazyme 15
Reference List 166
Monitoring scheme 177

Summary "Treatment of Fabry patients > 18 years with enzyme supplementation therapy: comparison of efficacy and toxicity of low dose (0,2 mg/kg) Fabrazyme (agalsidase beta) or Replagal (agalsidase alfa)"

Introduction	Fabry disease is an X-linked disorder caused by the deficiency of the lysosomal enzyme αGalactosidase A (α-Gal). Patients with this disorder suffer in childhood from severe pains in hands and feet and develop severe complications later in life such as renal failure, CVA's and cardiac complications. Patients with Fabry disease have a reduced life expectancy. Recently two differently α-Galactosidase enzyme preparations have received marketing authorization in the EU (orphan drug). Conclusions on the differences between these products with regard to safety and efficacy cannot be drawn because of the different dosage and evaluation methods performed. Therefore treatment should be performed according to a standardized treatment approach which allows comparison of both drugs.
Protocol objectives	To monitor and evaluate the efficacy and safety of two different formulas of α-Galactosidase A, agalsidase alpha (Fabrazyme™) and agalsidase beta (Replagal™) in an equal dose of 0,2 mg/kg in adults with Fabry disease and to investigate the feasibility of home treatment.
Investigational plan	Symptomatic Fabry patients (aged 18 or older) who fulfil the criteria will receive enzyme therapy for a minimum of 12 months and will be evaluated at month 1,2,3,4,5,6, and every following 3 months.
Patients and duration	Patients must have Fabry disease and at least one major or two minor objective criteria:<br>Major:<br>1 Severe acroparesthesias, that cannot satisfactorily be controlled with carbamazepine<br>2 Decreased GFR < 80 ml/min<br>3 Proteinuria > 300 mg/24h<br>4 Documented CVA<br>5 Cardiac infarction<br>6 Hypertrophic non-obstructive cardiomyopathy resulting in decreased exercise tolerance.<br>7 Rhythm disturbances necessitating a pacemaker<br>8 Multiple lacunar infarctions on MRI.<br>Minor:<br>1. Documented TIA.<br>2. Cardiac hypertrophy on echo or MRI.<br>3. Atrial fibrillation.<br>4. Intraventricular conduction abnormality.<br>5. Sensoric hearing loss as shown on a hearing test.<br>6. Severe vertigo<br>7. Micro-albuminuria (> 50mg/24h)<br>8. Mild to moderate acroparesthesias<br>9. Gastrointestinal complaints that cannot be explained by medical conditions other than Fabry disease.
Treatments	Patients will receive either 0.2 mg/kg Fabrazyme or 0.2 mg/kg Replagal biweekly for a minimum of 12 months.
Risks	No additional assessments will be performed than are done for normal standard care for patients with Fabry disease who will start enzyme supplementation therapy.
Efficacy, safety and sample size	Primary endpoint:<br>Reduction of left ventricular mass on echocardiography<br>Secondary endpoint:<br>• Improvement of renal function as measured by GFR.<br>• Reduction of glycolipid accumulation in skin tissue (LM and biochemistry).<br>• Reduction in pain as measured by the BPI.<br>• Reduction in glycosphingolipid in plasma and 24-hour urine.

	Tertiary endpoint:<br>• Quality of life scores (SF-36).<br>• Treatment failure defined as<br>- Progression of renal disease (33% increase in serum creatinin, need for dialysis or transplantation),<br>- Progression of cardiac disease (new infarction, need for cardioversion, or anti-arrythmic drugs, heart-failure necessitating hospitalization)<br>- Occurrence of a new CVA as diagnosed by a neurologist or new lacunar infarctions on magnetic resonance imaging (MRI) as assessed by an experienced neuroradiologist

INTRODUCTION

Fabry disease is an X-linked disorder caused by the deficiency of the lysosomal enzyme alpha Galactosidase A (α-Gal). Due to this deficiency specific glyoclipids accumulate in the entire body, but predominantly in the vascular endothelial cells. Males with this disorder suffer in childhood from severe pains in hands and feet (acroparesthesias), hypo- or anhidrosis and have specific skin lesions at the trunk and genitals, so-called angiokeratoma. Later in life, they develop complications due to the accumulation of glycolipid in endothelial cells: renal failure, cardiomyopathy (cardiac hypertrophy and rhythm disturbances) and cerebro-vascular accidents (1;2). Originally, female carriers were believed to be asymptomatic, but symptomatic females (even with end-stage renal failure) have been described and are also known within the Dutch population. Of the 70 currently known carriers in the Netherlands about 5 exhibit clinical complications due to Fabry disease, mostly moderate renal failure or stroke. More than 100 different mutations resulting in α-Gal deficiency have already been described. Currently in the Netherlands 22 different mutations are known.

Fabry disease is a debilitating disorder: the painful episodes in childhood and adolescence are followed by an increasing morbidity due to the vascular complications arising from the endothelial accumulation of lipids. Until recently only symptomatic treatment such as pain management, dialysis or renal transplantation was available. Lately, two studies have shown that enzyme supplementation therapy can reduce or effectively clear the amount of accumulated glycosphingolipids in renal, cardiac or skin tissue(3;4). It is believed that reducing or clearing the amount of accumulated lipid can halt disease-progression. In the mentioned studies different enzyme preparations have been investigated, Agalsidase alpha (Replagal™ produced by TKT, Inc.) and Agalsidase beta (Fabrazyme™ produced by Genzyme corp.). Both products have received a positive opinion of the CPMP in March which has resulted in a market authorization in August 2001.

Background of the efficacy protocol:
As stated, the effectivity and safety of Fabrazyme and Replagal have been evaluated with different methods. Therefore, a preference of one enzyme preparation over the other for the treatment of Fabry-patients cannot be established. This has led to the approval of both products, whereas normally only one Orphan Drug receives marketing approval. Apart from the difference in evaluation methods, both product have been administered in different dosing regimens: Fabrazyme at a dose of 1,0 mg/kg/14 days and Replagal at a dose of 0,2 mg/kg/14 days. This hampers determination of the most effective treatment. To be able to treat Fabry patients optimally, it is important to gain insight in the difference of both products. This protocol has been developed to evaluate both enzyme products in an uniformal way and at the same dose.

Detailed biochemical studies with Fabrazyme and Replagal at the department of Biochemistry (prof. dr. J.M.F.G. Aerts) did not reveal any significant difference between the two preparations with regard to chemical composition, enzyme-activity, stability, and uptake and kinetics in cultured Fabry fibroblasts. In a Phase I study, Fabrazyme was evaluated at different dosage regimens: 0.3, 1.0 en 3.0 mg/kg, for five infusions(5). Patients receiving 0,3 mg/kg also demonstrate a significant reduction in glycolipid accumulation in liver, skin and plasma. Replagal has not been tested in Phase I studies at higher dosage regimens than 0,2 mg/kg(6). Because of the absence of in vitro differences between these enzyme products a

clinical study in which both enzyme preparations will be administered at a dosage regimen of 0,2 mg/kg seems justified.

This treatment protocol aims to evaluate in a uniformal way the safety and efficacy of treatment of adult Fabry patients with Agalsidase alpha or beta.

PROTOCOL OBJECTIVES

The objectives of the protocol are the following:

To monitor and evaluate the efficacy and safety of two different formulas of α-Galactosidase A, agalsidase beta (Fabrazyme^{TM}) and agalsidase alpha (Replagal^{TM}) in an equal dose of 0,2 mg/kg.

To evaluate the feasibility of home treatment.

INVESTIGATIONAL PLAN

Study design and scheme

Symptomatic and biochemically proven Fabry patients fulfilling the inclusion criteria, both male and female, will be eligible. They will receive training in infusing the medication, at a dose of 0,2 mg/kg (Agalsidase alpha or beta). Patients will be monitored at baseline and month 1,2,3,4,5,6,and every following three months of treatment.

Duration of treatment protocol.

The duration of this treatment protocol will be at least 12 months. After 12 months of treatment the study will be continued for an additional12 to 24 months, using the same endpoints.

PATIENT POPULATION AND SELECTION

Criteria for treatment

Patients must have the following criteria to be enrolled in this study:

1. The patient must have written informed consent when the drug is provided on a compassionate use basis.
2. Patients must be 18 years or older.
3. Patient must have a current diagnosis of Fabry disease.
4. Patients must have a decreased α-Gal activity or proven α-Gal A mutation.
5. Female patients must have a negative pregnancy test, and must use a medically accepted method of contraception.
6. Patients must be willing to comply to the evaluation program.
7. Patients must have a clinical presentation consistent with either typical or atypical Fabry disease. Patients must have at least one major or two minor objective criteria:
   Major:

1 Severe acroparesthesias, that cannot satisfactorily be controlled with Carbamazepine
2 Decreased GFR < 80 ml/min
3 Proteinuria > 300 mg/24h
4 Documented CVA
5 Cardiac infarction
6 Hypertrophic Non-obstructive Cardiomyopathy resulting in decreased exercise tolerance.
7 Rhythm disturbances necessitating a pacemaker
8 Multiple lacunar infarctions on MRI.

Minor:
1. Documented TIA.
2. Cardiac hypertrophy on echo or MRI.
3. Atrial fibrillation.
4. Intraventricular conduction abnormality.
5. Sensoric hearing loss as shown on a hearing test.
6. Severe vertigo.
7. Micro-albuminuria > 50 mg/24h.
8. Mild to moderate acroparesthesias
9. Gastrointestinal complaints that cannot be explained by medical conditions other than Fabry disease.

Note: Angiokeratoma and cornea verticillata are NOT criteria for treatment.

Exclusion criteria
1. Patient is pregnant or lactating.
2. Patient is unwilling to comply to the evaluation program.

Patient withdrawal
Patients will be excluded from treatment if the following occurs:
1. Intolerability of the drug, shown by infusions related serious adverse events as judged by the treating physician.
2. Concomitant severe disease resulting in very short life expectancy, or the inability to administer the treatment.

TREATMENTS

Treatments administered
Patients will receive 0,2mg/kg Fabrazyme or 0,2 mg/kg Replagal, every two weeks. Patients will be randomised to receive either Fabrazyme or Replagal 0,2 mg/kg.

Randomization
Randomisation codes will be generated and envelops sealed by an independent person using permuted block randomisation, with blocksize of four patients. Upon entry in the study the participating patient will draw an envelope, in which is denoted what enzyme product that patient will receive.

Administration of low-dose agalsidase (Fabrazyme or Replagal 0,2 mg/kg)
Patients will be learned how to infuse themselves with r-hαGal during s short-stay at the Academic Medical Centre, by trained nurses. Infusions will be prepared by the patients

following a detailed training at the AMC. Preparation is done in accordance to the manufacturer's recommendations. The drugs will be infused in ~40 minutes infusion, followed by a short wash-out with NaCl. Patients will receive the first treatment at the AMC and will be treated at a local hospital for the next 6 months. After that period, the patient will be given the possibility to continue treatment at home, when the treating physician feels it is safe. Back-up during home treatment will be organized on an individual basis. The possibility of immediate help by a local physician or nurse should be provided. At every study visit the results of home treatment will be recorded.

Prior and concomitant Medications
Prior to infusion no medication will be given. Concomitant medication will be continued or changed by discretion of the treating physician. All changes in medication will be recorded.

Investigational products

Reports from the Committee For Proprietary Medicinal Products (CPMP) of the European Agency for the Evaluation of Medicinal Products (EMEA). Summary of Opinion, Londen 29 March 2001.

Fabrazyme
International Non-proprietary Name (INN): agalsidase beta.

On 29 March 2001 the Committee For Proprietary Medicinal Products (CPMP) adopted a positive opinion to recommend the granting of a marketing authorisation for the medicinal product Fabrazyme 35 mg powder for concentrate for solution for infusion intended for long-term enzyme replacement therapy in patients with Fabry disease. Fabrazyme was designated as an orphan medicinal product on 8 August 2000. The applicant for this medicinal product is Genzyme B.V.

The active substance of fabrazyme is agalsidase beta, a recombinant α-galactosidase A produced by genetically engineered Chinese Hamster Ovary (CHO) cells.

The benefits with Fabrazyme in this glycosphingolipid storage disorder include the reduction or clearance of glycosphingolipids in the plasma and cells of the kidney, heart and skin. Renal function remained stable and a stabilisation of neuropathic pain was observed with treatment up to one year. These results are suggestive of a clinical improvement or a stabilisation of the clinical condition. The available data indicate an acceptable safety profile for Fabrazyme. The most common side effects are hypersensitivity reactions, which were manageable with appropriate interventions. The number of patients treated is relatively small and the treatment duration in the clinical trials relatively short compared to the long-term treatment necessary for Fabry disease. However, due to the rarity of the condition and the evidence provided to support the rationale for a beneficial effect of enzyme replacement therapy, it is considered acceptable that additional data will be submitted by the applicant as post-marketing obligations. These long-term safety and efficacy data shall form the basis of the annual reassessment of the benefit/risk ratio profile for Fabrazyme.

The approved indication is: long-term enzyme replacement therapy in patients with a confirmed diagnosis of Fabry disease (α-galactosidase deficiency). Fabrazyme treatment should be supervised by a physician experienced in the management of patients with Fabry Disease or other inherited metabolic diseases. Detailed conditions for the use of this product will be described in the Summary of Product Characteristics (SPC) which will be published in

the European Public Assessment Report (EPAR) and will be available in all official Europena Union languages after the marketing authorization has been granted by the European Commission.

The CPMP, on the basis of quality, safety and efficacy data submitted, considers that there is a favourable benefit to risk balance for Fabrazyme and therefore recommends the granting of the marketing authorization under exceptional circumstances.

Replagal
International Non-proprietary Name (INN): agalsidase alfa (pending).

On 29 March 2001 the Committee For Proprietary Medicinal Products (CPMP) adopted a positive opinion to recommend the granting of a marketing authorisation for the medicinal product Replagal 1mg/ml concentrate for solution for infusion intended for long-term enzyme replacement therapy in patients with Fabry disease. Fabrazyme was designated as an orphan medicinal product on 8 August 2000. The applicant for this medicinal product is TKT Europe-5S AB.

The active substance of Replagal is agalsidase alfa, a human α-galactosidase A produced by genetically engineering technology in a human cell line..

The benefits with Replagal in this glycosphingolipid storage disorder include the reduction of neurpathic pain, initial stabilisation followed by improvement of renal function with long-term use and a reduction in cardiac mass. A reduction of glycosphingolipid content in the plasma, urine sediment and cells of the kidney, heart and liver was also observed. These results are suggestive of an improvement of the clinical condition with continued Replagal therapy. The available data indicate an acceptable safety profile for Replagal. The most common side effects are infusion reactions, which were manageable with appropriate interventions. The number of patients treated is relatively small and the treatment duration in the clinical trials relatively short compared to the long-term treatment necessary for Fabry Disease. However, due to the rarity of the condition and the evidence provided to support the rationale for a beneficial effect of enzyme replacement therapy, it is considered acceptable that additional data will be submitted by the applicant as post-marketing obligations. These long-term safety and efficacy data shall form the basis of the annual reassessment of the benefit/risk ratio profile for Replagal.

The approved indication is: long-term enzyme replacement therapy in patients with a confirmed diagnosis of Fabry disease (α-galactosidase deficiency). Replagal treatment should be supervised by a physician experienced in the management of patients with Fabry Disease or other inherited metabolic diseases. Detailed conditions for the use of this product will be described in the Summary of Product Characteristics (SPC) which will be published in the European Public Assessment Report (EPAR) and will be available in all official Europena Union languages after the marketing authorization has been granted by the European Commission.

The CPMP, on the basis of quality, safety and efficacy data submitted, considers that there is a favourable benefit to risk balance for Replagal and therefore recommends the granting of the marketing authorization under exceptional circumstances.

Packaging
The Agalsidases will be kept in a secure place and stored at 2 to 8 C. Patients are asked to keep their vials when used and to return them on a study-visit. Records will be kept of all medications received, administered and returned.

Reconstitution
Fabrazyme is supplied in 20 ml vials (35 mg/vial). Reconstitution will take place with 7.2 ml of sterile water for injection. The appropriate amount of enzyme needed for infusion is further diluted with 0.9% sodium to a final volume of 100 ml in the case of a dose of 0,2 mg/kg.

Replagal is supplied in 10 ml vials (3.5 mg/vial in 3.5 ml saline). No reconstitution is required and The appropriate amount of enzyme needed for infusion is will be diluted with 0.9% sodium to a final volume of 100 ml.

Reimbursement
Reimbursement of Fabrazyme and Replagal is covered by a subsidiairy from the Durch Ministry of Health (VWS) both for the in and out-hospital use. The pharmaceutic department at the AMC (drs. P Langendijk) is the coordinator of the distribution in the Netherlands.

Evaluation and monitoring of treatment

Baseline:
The following assessments have to be performed prior to the first infusion.

- Safety labs
- Physical assessment
- Neurologic assessment **)
- Quality of Life (SF-36)
- Pain-scores (Brief Pain Inventory)
- EKG
- Echocardiography
- MRI / MRS brain **)
- Skin biopsy
- GFR
- Hearing test + evaluation by ENT-specialist
- 24hour urine:
  - protein
  - Kreatinin
  - Globotriaosylceramide
- Plasma
  - A-Gal antibody titer
  - Globotriaosylceramide
  - Kreatinin
  - Hydrolase

Post-infusion α-Gal activity
After the first infusion with α-Gal A blood samples will right after the end of the infusion and subsequently at 60 and 120 minutes for determination of α-Gal A activity.

Month 1,2

- Safety labs
- Plasma
  - A-Gal antibody titer
  - Globotriaosylceramide

Month 3 and every following 3 months

- Safety labs
- EKG
- Physical examination
- 24hour urine:
  - protein
  - Kreatinin
  - Globotriaosylceramide
- Plasma
  - A-Gal antibody titer
  - Globotriaosylceramide
  - Kreatinin
  - Hydrolase

Month 6, 12, 24

- Safety labs
- Neurologic assessment **)
- Quality of Life (SF-36)
- Pain score
- EKG
- Echocardiography
- MRI / MRS brain **)
- Skin biopsy
- GFR
- Hearing test + evaluation by ENT-specialist
- 24hour urine:
  - protein
  - Kreatinin
  - Globotriaosylceramide
- Plasma
  - A-Gal antibody titer
  - Globotriaosylceramide
  - Kreatinin
  - Hydrolase

**) Optional, by discretion of the treating physician.

EFFICACY AND SAFETY VARIABLES

All laboratory tests will be performed at the local institute. Only the skin biopsy, 100 mL of urine, plasma for glycolipid analysis and hydrolase activity will be shipped to and performed at the AMC (department of biochemistry).

Evaluation of GFR
All patients will have a GFR-test. In the AMC the test as described by Appelloo et al. (125I-iothalamate and 131I-hippuran) with some minor adjustments in calculation is used.(7)

Evaluation of skin tissue
Two 3mm punches of skin will be obtained prior to and after 6 and 12 months of study. One sample will be used for assessment of lipid-accumulation by a blinded pathologist. The other sample will be used for biochemical assessment of glycolipid.

Pain-scores (BPI) and quality of life (SF-36)
The Brief Pain Inventory and the SF-36 will be obtained at Baseline, Month 6, 12 and 24 of treatment.

Urinary Kreatinin and protein
24 Hour urine will be collected at home by the patient. Protein and Kreatinin levels will be determined at the local institute. 100 mL of 24-hour urine will be frozen and shipped to the AMC for glycolipid analysis.

Urinary Glycolipid
Determiniation of glycolipid-levels in 100 mL of 24-hour urine is performed at the biochemistry department at the AMC using a specific and unique HPLC-method.

Plasma Glycolipid
Plasma will be obtained at baseline and month 1,2,3,4,5,6,and every following 3 months. Determination of glycolipid-levels in plasma is performed at the biochemistry department at the AMC using a specific and unique HPLC-method.

Physical examination
Is performed by the treating physician experienced in Fabry disease. All clinically significant changes will be noted and recorded.

Electrogradiogram
A standard ECG will be performed. Rate, rhythm, intervals, axis, conduction abnormalities and overall cardiac impression of each patient will be recorded.

Echocardiogram
A standard 2-dimensional Doppler echocardiogram will be performed and will include ventricular cavity size, ventricular wall thickness, valvular abnormalities and estimated ejection fraction. .

Safety lab assessments
Serum chemistry , haematology and urinalysis will be performed as safety measurements. The following will be measured:

- CBC, with differentiation.
- BUN, Kreatinin, Glucose, Uric acid, calcium, phosphorus, albumin, total protein, sodium, potassium, chloride, bicarbonate, SGOT. SGPT, total bilirubin, alkalin phosphatase
- Routine urinalysis.

Academic Medical Center, December 2004.

Antibody testing
Blood serum samples will be obtained for the detection and quantification of antibodies against agalsidase alpha or beta.

MRI assessments
A brain MRI will be performed at baseline and after 12 and 24 months of treatment, and will serve as a safety measurement.

Hearing test
All patients must undergo a routine hearing-test at the local facility and an evalution by an ENT-specialist before and after 12 and 24 months of treatment.

Adverse and serious adverse experiences
Any adverse events of serious adverse events will be noted.

Statistical methods and planned analyses

Efficacy endpoints:
The following endpoint wills serve to evaluate the safety and efficacy of Agalsidase alpha and beta in the treatment of patients with Fabry disease. In addition these endpoints will also be used to detect any differences between these two drugs.

Left ventricular hypertrophy is an important complication seen in Fabry disease, which influences morbidity and mortality. As agalsidase alpha therapy has shown to significantly reduce left ventricular mass in case of cardiac hypertrophy (8) this has been chosen to serve as a primary endpoint.

Primary endpoint:

- Reduction of left ventricular mass on echocardiograpy.

Secondary endpoint:

- Improvement of renal function as measured by GFR.
- Reduction of glycolipid accumulation in skin tissue (LM).
- Reduction of glycolipid accumulation in skin tissue (biochemistry).
- Pain-scores
- Reduction in glycosphingolipid in plasma and 24-hour urine.

Tertiary endpoint:

- Quality of life scores (SF-36).
- Treatment failure
  If during the course of the study a patient shows progression of disease he will be advised to switch to treatment with agalsidase beta 1,0 mg/kg biweekly.
  Definition of treatment failure:
  - Progression of renal disease (33% increase in serum creatinin, need for dialysis or transplantation),
  - Progression of cardiac disease (new infarction, need for cardioversion, or anti-arrythmic drugs, heart-failure necessitating hospitalization)

- Occurrence of a new CVA as diagnosed by a neurologist or new lacunar infarctions on magnetic resonance imaging (MRI) as assessed by an experienced neuroradiologist

Safety endpoints:

- Safety labs
- EKG
- Stabilization or improvement in MRI / MRS
- Echocardiography
- Physical examination
- Measurement of r-hαGal antibodies
- Adverse experiences
- Serious adverse events

Efficacy analysis
Summary tables will show: n, mean, st.dev, median and range for all efficacy variables and its change score at the different times measured. Statistical analyses will be performed to show any differences in changes of several variables between the two treatment-groups (preferably non-parametric).

Sample size estimation to detect a possible difference between Replagal and Fabrazyme
The estimated sample size necessary to perform a comparison of the two treatmet groups for reduction of left ventricular mass has been calculated. Previous studies mentioned a reduction of 20% in Fabry patients with cardiac hypertrophy following 12 months of agalsidase alfa (0.2 mg/kg) treatment (8). Agalsidase beta 1.0 mg/kg treatment, however, only resulted in a 10% reduction of left ventricular mass as assessed by MRI (9)
The assumption (null-hypothesis) is that there is a more pronounced reduction of left ventricular mass (of at least 10%) on agalsidase alfa than agalsidase beta after 12 months of treatment. With the following assumptions (estimated sd of the change in left ventricular mass 5.5%- 7.5%, power of 90% and statistical significance of <0,05) one can conclude that 18 patients (with an increased left ventricular mass) have to be available to detect a difference.

Academic Medical Center, December 2004.

Reference List

(1) Desnick RJ, Ioannou YA, Eng ME. a-Galactosidase A Deficiency: Fabry disease. in: Scriver CR, Beaudet AL, Sly WS, Valle D, eds The metabolic and molecular bases of inherited disease 6th ed New York: McGraw-Hill 1996;2741-2784.

(2) Linthorst GE, Hollak CEM, Bosman DK, Heymans HSA, Aerts JM. De ziekte van Fabry: op weg naar een behandeling. Ned Tijdschr Geneeskd 2000; 144(50):2391-2395.

(3) Schiffmann R, Kopp JB, Austin HA, III, Sabnis S, Moore DF, Weibel T et al. Enzyme replacement therapy in fabry disease: a randomized controlled trial. JAMA 2001; 285(21):2743-2749.

(4) Eng CM, Guffon N, Wilcox WR, Germain DP, Lee P, Waldek S et al. Safety and efficacy of recombinant human alpha-galactosidase A-- replacement therapy in Fabry's disease. N Engl J Med 2001; 345(1):9-16.

(5) Eng CM, Banikazemi M, Gordon RE, Goldman M, Phelps R, Kim L et al. A Phase 1/2 Clinical Trial of Enzyme Replacement in Fabry Disease: Pharmacokinetic, Substrate Clearance, and Safety Studies. Am J Hum Genet 2001; 68(3):711-722.

(6) Schiffmann R, Murray GJ, Treco D, Daniel P, Sellos-Moura M, Myers M et al. Infusion of alpha-galactosidase A reduces tissue globotriaosylceramide storage in patients with Fabry disease. Proc Natl Acad Sci U S A 2000; 97(1):365-370.

(7) Apperloo AJ, de Zeeuw D, Donker AJ, de Jong PE. Precision of glomerular filtration rate determinations for long-term slope calculations is improved by simultaneous infusion of 125I- iothalamate and 131I-hippuran. J Am Soc Nephrol 1996; 7(4):567-572.

(8) Beck M, Ricci R, Widmer U, Dehout F, de Lorenzo AG, Kampmann C et al. Fabry disease: overall effects of agalsidase alfa treatment. Eur.J Clin.Invest 2004;34(12):838-44.

(9) Weidemann F, Breunig F, Beer M, Sandstede J, Turschner O, Voelker W et al. Improvement of Cardiac Function During Enzyme Replacement Therapy in Patients With Fabry Disease. A Prospective Strain Rate Imaging Study. Circulation 2003:01.

Academic Medical Center, December 2004.

Monitoring scheme

Assessment	Baseline	#1 infusion	#2 infusion	Month 2,4,5	Month 3	Month 6	Month 9	Month 12	Every 3rd month	Every 6th month
Informed consent	X	[redacted]								
Enzyme activity *	X	[redacted]								
Mutation analysis *	X	[redacted]								
Pain score	X				X	X	X	X	X	X
Quality of Life	X					X		X		X
24 hr urine * Kreatinin	X				X	X	X	X		X
* GL-3	X				X	X	X	X		X
GFR	X					X		X		
Plasma GL-3	X		X	X	X	X	X	X		X
A-Gal Antibodies		X	X	X	X	X	X	X	X	X
Skin biopsy	X					X		X		
MRI / MRS	X							X		
IMT measurement	X							X		
ENT-evaluation	X							X		
Safety labs / urinalysis	X	X	X		X	X	X	X	X	X
EKG	X					X		X		X
Echocardiography	X					X		X		X
Neurological assessment	X							X		
Hydrolase-activity	X	X	X	X	X	X	X	X	X	X

* If not already performed.
